# Molecular Regulation of Bone Turnover in Juvenile Idiopathic Arthritis: Animal Models, Cellular Features and TNF*α*

**DOI:** 10.31083/j.fbl2907248

**Published:** 2024-07-10

**Authors:** Harry C Blair, Jonathan Soboloff, Irina L Tourkova, Jamie L. McCall, Suravi Ray, Margalit E Rosenkranz, Cristina Sobacchi, Lisa J Robinson, John B Barnett

**Affiliations:** 1 Departments of Pathology and of Cell Biology, The Pittsburgh VA Medical Center and the University of Pittsburgh, Pittsburgh, PA 15261, USA; 2 Department of Cancer and Cellular Biology, Fels Cancer Institute for Personalized Medicine, The Lewis Katz School of Medicine at Temple University, Philadelphia, PA 19140, USA; 3 Department of Microbiology, Immunology & Cell Biology, West Virginia University School of Medicine, Morgantown, WV 26506, USA; 4 *ExesaLibero* Pharma, Morgantown, WV 26505, USA; 5 Division of Rheumatology, UPMC Children’s Hospital of Pittsburgh, Pittsburgh, PA 15224, USA; 6 National Research Council (CNR)-Institute of Genetic and Biomedical Research (IRGB), Milan Unit, 20138 Milan, Italy; 7 IRCCS Humanitas Research Hospital, 20089 Rozzano, Italy; 8 Department of Pathology, Nebraska Medical Center Omaha, Omaha, NE 68198, USA

**Keywords:** rheumatoid arthritis, juvenile idiopathic arthritis, macrophage, osteoclast, TNF*α*, interleukin 1, interleukin 6

## Abstract

We review the abnormal bone turnover that is the basis of idiopathic inflammatory or rheumatoid arthritis and bone loss, with emphasis on Tumor Necrosis Factor-alpha (TNF*α*)-related mechanisms. We review selected data on idiopathic arthritis in juvenile human disease, and discuss mouse models focusing on induction of bone resorbing cells by TNF*α* and Receptor Activator of Nuclear Factor kappa B Ligand (RANKL). In both humans and animal models, macrophage-derived cells in the joint, particularly in the synovium and periosteum, degrade bone and cartilage. Mouse models of rheumatoid arthritis share with human disease bone resorbing cells and strong relation to TNF*α* expression. In humans, differences in therapy and prognosis of arthritis vary with age, and results from early intervention for inflammatory cytokines in juvenile patients are particularly interesting. Mechanisms that contribute to inflammatory arthritis reflect, in large part, inflammatory cytokines that play minor roles in normal bone turnover. Changes in inflammatory cytokines, particularly TNF*α*, are many times larger, and presented in different locations, than cytokines that regulate normal bone turnover. Recent data from *in vitro* and mouse models include novel mechanisms described in differentiation of bone resorbing cells in inflammatory arthritis dependent on the Transient Receptor Potential Channel (TRPC) family of calcium channels. Low-molecular weight (MW) inhibitors of TRPC channels add to their potential importance. Associations with inflammatory arthritis unrelated to TNF*α* are briefly summarized as pointing to alternative mechanisms. We suggest that early detection and monoclonal antibodies targeting cytokines mediating disease progression deserves emphasis.

## 1. Introduction

### 1.1 Bone Damage in Juvenile Idiopathic Arthritis

Inflammatory arthritis is the most common inflammatory disease associated with skeletal joint bone destruction; known as juvenile idiopathic arthritis in children or rheumatoid arthritis in adults. To emphasize common pathology, we refer to juvenile idiopathic arthritis or rheumatoid arthritis as inflammatory arthritis. It is impractical to discuss all aspects of the diseases; we consider mainly improved outcomes in juvenile idiopathic arthritis.

Juvenile idiopathic arthritis is inflammatory arthritis generally defined as occurring by age 16 [1]. There is no basis for the sharp cutoff in age, but it is convenient to avoid confusion. Rheumatoid arthritis predominately affects women over age 40 [2]. In either case, the arthritis has high levels of inflammatory molecules especially tumor necrosis factor-alpha (TNF*α*), but also interleukins 1 and 6 (IL-1 and IL-6). Children responding to biological inhibitors such as anti-TNF*α*, IL-1 or IL-6 have extremely promising outcomes [3], better than those in adults. However, children with inflammatory arthritis present at earlier stages than adult patients, which is a probable factor in outcomes.

### 1.2 The Structure of the Rheumatoid Joint is Shown Using Animal Models

Many people who work with rheumatoid arthritis rarely see its histology; this is impractical in human disease. We compare arthritic joint damage, with very dense predominately mononuclear infiltrates, with normal bone turnover, which is distinct, lacking the infiltrates.

### 1.3 Changes in Cytokine Expression in the Rheumatoid Joint are Complex

Inflammatory cytokines, particularly in TNF*α*, increase hundreds of folds. This leads to many secondary responses. An important example is that soluble receptor activator of NF-*κ*B (RANKL) increases ~50% inflammatory arthritis. TNF*α* is a stimulus for NF-*κ*B, a property often overlooked.

### 1.4 Limitations of this Review

This is a selective review, a significant limitation, but a practical approach to allow cogent discussion. There are far too many publications on Juvenile Idiopathic Arthritis to review all, and no standard format for studies of Juvenile Idiopathic Arthritis, making a systematic comparison impractical. Similarly, studies in each section cannot logically be listed in tabular format. However, each subject was searched and all relevant publications were evaluated. Fine points of other factors in etiology are not considered; they are irrelevant to juvenile disease. These include antibodies to cyclic citrullinated proteins in the progression of adult disease. Citrullinated proteins are released by apoptotic cells, and in adult disease, antibodies are important indicators of progression. Similarly, nonspecific metabolic inhibitors are not considered in detail. Additionally, we show detailed features of animal models of rheumatoid arthritis, which are believed to reflect in major part features of human disease, which have limitations but which cannot ethically be studied in humans with developing arthritis.

## 2. Cytokines and Related Cellular Chages in Juvenile Idiopathic Arthritis

### 2.1 Cytokines and Antibodies in Arthritis Progression, with Emphasis on Juvenile Disease

In juvenile idiopathic arthritis, cell-free synovial fluid shows large increases in TNF*α*, IL-6, IL-10, interferon-*γ*, and IL-17 [4], which include cytokines that stimulate osteoclast formation independently of RANKL [5]. There are similar data on rheumatoid arthritis in adults [6]. There is some overlap with inflammatory factors in other arthritides: TNF*α*, IL-6, and IL-17 are greatly increased in, though not specific for, inflammatory arthritis. In adults, first level therapy has been the antimetabolite methotrexate and steroids, typically prednisone. Alternatively, due to an increase in inflammatory cytokines in synovial fluid, humanized antibodies, including anti-TNF*α*, may produce a remarkable response [7]. Prior to the availability of anti-TNF*α* therapy, inflammatory arthritis in children often required joint replacement [8,9], now unusual in our experience.

This has not escaped the attention of adult rheumatologists, and it is generally believed that anti-TNF*α* therapy may at least in part prevent development of auto-antibodies related to progression of disease [10–12]. A key problem with preventing erosive joint damage in adult rheumatoid arthritis is that erosive joint damage is common when diagnosis of Rheumatoid arthritis (RA) is made [11]. How earlier detection might be achieved, possibly by screening for elevated cytokines in at-risk patients, is not clear. Screening x-rays are usually not done in adolescents; erosive damage at diagnosis is uncommon.

### 2.2 Cellular Features of Inflammatory Arthritis

**Fig. 1.**
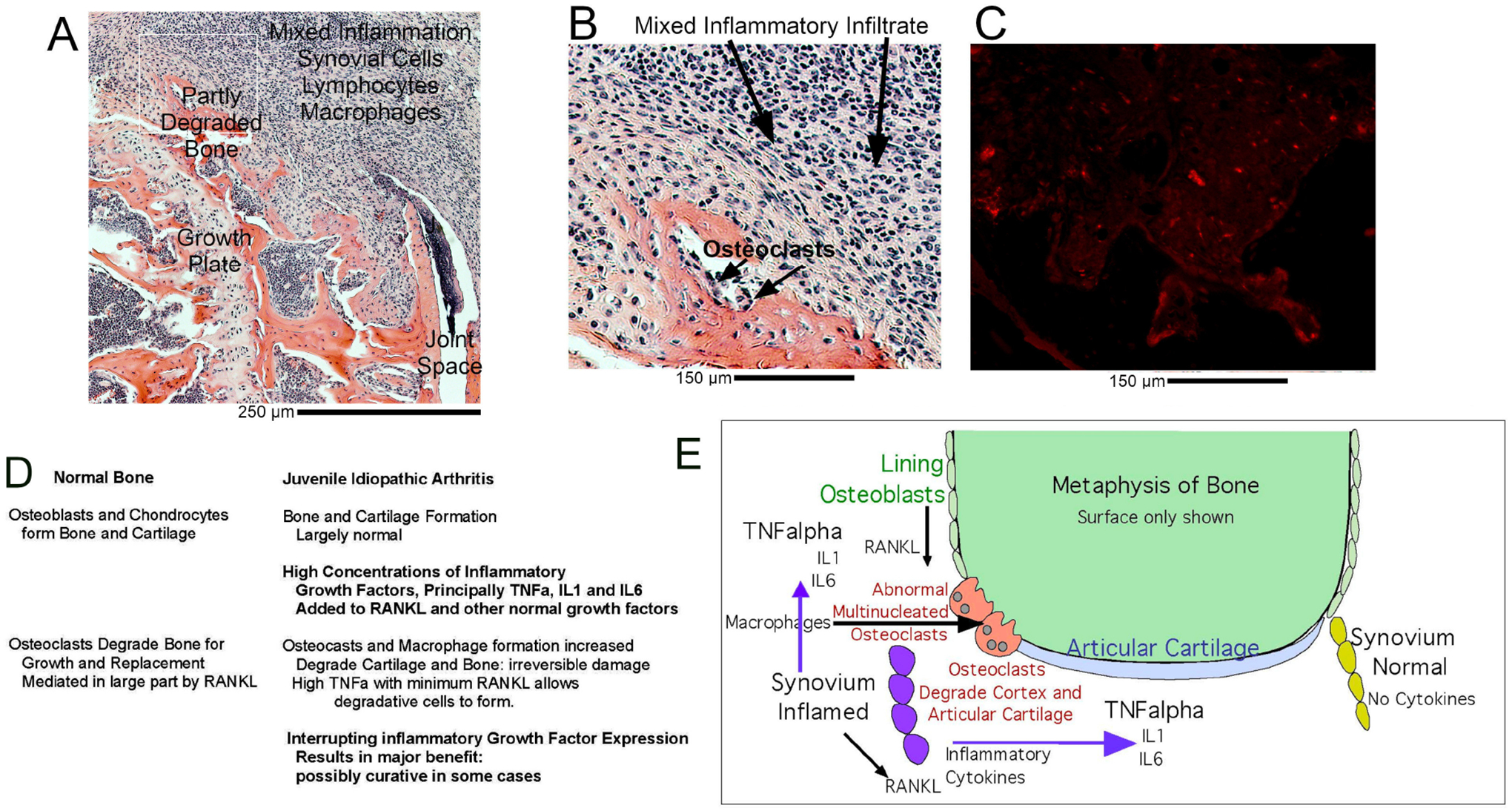
Inflammatory arthritis with degradation of bone by surrounding tissue illustrated as a composite of unpublished images of sections from [13] of collagen-induced arthritis in mice. The contrast with orderly turnover and induction of osteoblasts in bone (Fig. 2 below) is striking. Reference [14] shows characterization of cells in more detail. Animal work was carried out with approval of institutional animal care and use committees protocol 1706006784 and others given in references quoted. (A) Mouse knee with an inflammatory synovium surrounding the joint. The expanded inflammatory synovium includes mixed lymphocytes and dense macrophages, as well as synoviocytes of mesenchymal cell origin, cf. [14]. The growth plate, articular plate (largely degraded) and infiltrate (above the bone) are labeled. The image is 0.5 mm on a side, taken at 20×. Mouse biology has limitations as a model of Juvenile Inflammatory Arthritis in humans, but shows clearly main features of the human disease. Scale bar: 250 μM. (B) A portion of the image (Fig. 2A), indicated by the square, at higher power; what is shown is 200 × 300 μm. The cells present include lymphocytes, synoviocytes of mesenchymal cell origin, and macrophage family cells, see [14]. At the site of bone degradation multinucleated cells occur, labeled osteoclasts. In any case, bone is degraded. Scale bar: 150 μM. (C) A similar section to (Fig. 2B) showing the pattern of tartrate-resistant acid phosphatase (TRAP) development in the inflammatory infiltrate above the bone by fluorescent TRAP labeling (red). Focal cells in a stippled pattern express TRAP, as well as larger osteoclasts on the bone (which appears dark, bottom of the section). Note that the formation of multinucleated cells is not solely bone associated as in normal bone turnover (Fig. 1A). Scale bar: 150 μM. (D) Summary diagram of key features of Juvenile Inflammatory Arthritis. Major inflammatory factors influencing the process are indicated. (E) Graphical diagram of main features of Juvenile Inflammatory Arthritis. Shown are the metaphysis (top) and epiphysis of a long bone (labeled only as articular cartilage for simplicity). In a normal bone (right) there is no inflammation and no abnormal amounts of inflammatory growth factors. In Juvenile Inflammatory Arthritis (left side) there is synovitis (inflammation of synovium) producing large amounts of tumor necrosis factor-alpha (TNF*α*) and in some cases interleukin (IL)-1 and IL-6. Small amounts of receptor activator of NF-*κ*B (RANKL) produced by cells in the joint normally do not support osteoclast formation, but in the presence of very large amounts of inflammatory growth factors multinucleated osteoclasts form from monocytic precursors on the bone cortex and cartilaginous surfaces, creating Juvenile Inflammatory Arthritis. Not all growth factors or cells are illustrated, but those shown are the principal mediators of the illness.

**Fig. 2.**
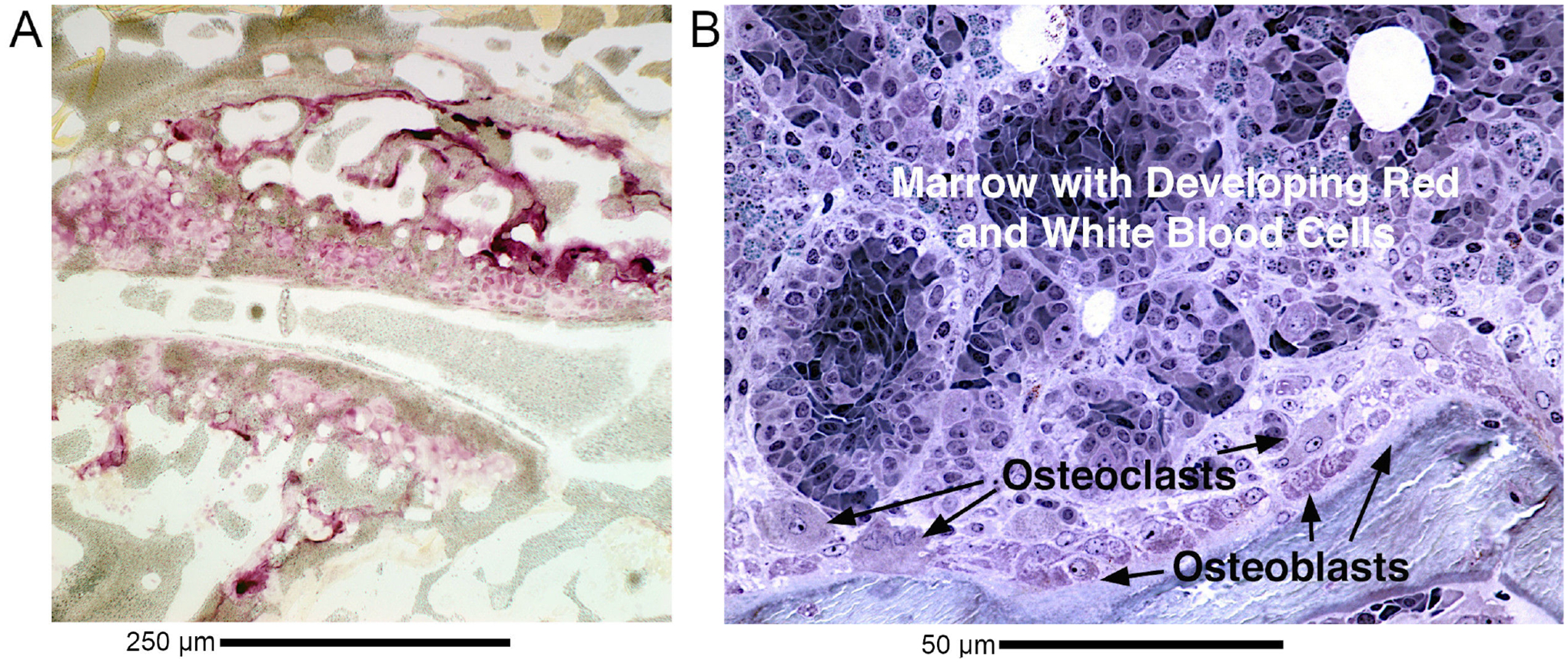
Osteoclasts forming directly adjacent to osteoblasts and mesenchymal cells around bone illustrated in sections of normal murine and avian bone. The images are not previously published, except for subimage A previously published as a control in Fig. 3C of Robinson LJ *et al*. [17], Laboratory Investigation. 2012; 92: 1071–1083. These provide a useful perspective on human bone formation and turnover, since it is not practical or ethical to do similar studies people, and are believed to reflect conserved biological features. Animal work was carried out with approval of institutional animal care and use committees protocol 1706006784 and others listed in references quoted. (A) Low power of a normal mouse bone joint in phase (grey) with fluorescent anti-TRAP (red) overlain to show the distribution of osteoclasts in a growing three-month-old mouse. Osteoclasts are seen in the bone lining, concentrated near the articular cartilages a site of high turnover. They are the bright red giant cells absent at other sites. The field is 500 μm wide. Fig. 2A is a section of a normal control animal [13,15]. Scale bar: 250 μM. (B) In normal bone, here avian bone, RANKL expressed on the cell surfaces drives multinucleation and differentiation of osteoclasts. Additional factors involved include colony-stimulating factor-1, which supports macrophage survival and is strongly expressed in the bone lining cells. Intracellular factors driving osteoclast differentiation include the nuclear factor of activated T-cells. The field shown is 100 μm wide. Scale bar: 50 μM. Fig. 2B is an avian bone embedded in plastic cut at 100 nm thickness, stained with methylene blue, and photographed in oil immersion optics at 100× [16].

These are illustrated in animal models, which are practical sources of high-quality sections. Note that animal models are limited, but provide a useful perspective where ethical use of human material is not possible. Adjacent to the joint, there is an extremely dense mononuclear inflammatory infiltrate, with apoptosis and degradation of bone and joint tissue. This is shown in Fig. 1 (Ref. [13,14]). The relationship between cartilage-degrading giant cells and osteoclasts is not settled, but the major issue remains, overall, control of joint damage. Destruction of bone and mineralized cartilage is by osteoclasts, multinucleated giant cells expressing tartrate-resistant acid phosphatase (TRAP). However, in inflammatory diseases, a key is that bone degradation may be sensitive to blocking the highly expressed inflammatory cytokines, especially TNF*α* [7], that mediate development of degradative cells in inflammatory infiltrates, at least in major part, as a secondary phenomenon. The contrast of inflammatory infiltrates surrounding joints to normal bone degradation by differentiation of osteoclasts within bone is discussed below and shown in Fig. 2 (Ref. [13,15–17]).

Juvenile idiopathic arthritis reflects still-unknown primary triggers for the immune system to target the joints. Unlike many autoimmune diseases, inflammatory arthritides are not mediated solely by autoantibodies. Autoantibodies occur mainly in long-term disease in adults. Antibodies to the Fc region of immunoglobulin G, “rheumatoid factor”, occur and include anti-citrullinated protein antibodies, which are also essentially specific for developing rheumatoid arthritis [18]. Citrullination of proteins in apoptotic cells [19] and anti-citrullinated protein antibodies are relevant mainly to adult disease including several autoimmune diseases [20].

Inflammatory arthritis is separate from osteo-arthritis, damage to cartilage due to trauma or overuse, and different from the spondyloarthropathies [21] associated with other diseases, e.g., inflammatory bowel disease and psoriasis in the presence of human leukocyte antigen B27 (HLA- B27). Therapy for spondyloarthropathies may overlap with management of inflammatory arthritis [22].

### 2.3 The Structure of the Rheumatoid Joint

Development of bone degrading cells, including multinucleated cells in inflammatory arthritis (Fig. 1), is in sharp contrast to osteoclasts developing in normal bone turnover (Fig. 2). Osteoclasts are multinucleated cells derived from monocyte-family precursors. Differentiation of osteoclasts is largely mediated by ligand for the RANKL, a product of cells of the mesenchymal lineage differentiating on the bone surface, which is the essential signal for normal osteoclast differentiation in bone turnover [23].

RANKL is also produced in lymphocytes and synovial cells, which are important in arthritis, e.g., [24]. The differentiation of osteoclasts also requires signaling by macrophage colony-stimulating factor (MCSF or CSF-1), by the nuclear factor of activated T-cells (NFATc), and by calcium signaling [25] including by the calcium-release activated calcium channel Orai1 [15]. More recently, it was discovered that a key additional signal is TRPC4, a member of the canonical subfamily of transient receptor potential cation channels. TRPC proteins forms calcium-permeable cation channels activated by Gq- coupled receptors or tyrosine kinases, involved in multiple processes outside of bone [26]. Our recent work points, in addition to the calcium-release activated calcium channel [13] to a specific role of TRPC4 in mediating formation of bone degrading cells.

Irrespective, osteoclast formation is, in normal bone, locally mediated. Osteoclasts differentiate from mononuclear precursors, apparently without intermediates at the site of bone turnover at the edge of the marrow (Fig. 1). We illustrate this with examples of bone in control animals from our recent work and high-resolution sections showing osteoclasts and osteoblasts in avian bone [16]. Key morphological features of osteoclasts include very high expression of tartrate-resistant acid phosphatase (TRAP) at the cell surface in bone, detectable as durable activity on bone when cells are stripped off bone matrix. Essential features of the osteoclast include massive surface expression of the V-type ATPase, which produces the acid needed to dissolve matrix of alkaline minerals. Mutations in the a3 subunit of the VATPase are the most common defect in human osteopetrosis [27]; other major functional molecules include a H^+^/Cl^*−*^ exchanger, the Chloride Voltage-Gated Channel 7 (ClC-7), and its *β*-subunit Ostm1 [28].

### 2.4 Cartilage and Bone Degrading Cells in Arthritic Joint Destruction

Characteristics of damage in inflammatory arthritis include expansion of synovium and periosteum and dense inflammatory cell infiltrates in the joint space. This creates a different environment for osteoclast differentiation and bone degradation. While the role of RANKL in osteoclast formation for bone turnover is established, it is also clear that inflammatory cytokines, including TNF*α*, IL-6 and IL-1, can promote non-canonical formation of multinucleated bone-degrading cells, in part independently of RANKL [5], though many workers believe that RANKL is essential and that inflammatory growth factors simply increase osteoclast formation locally. Additional inflammatory factors involved in promoting bone degradation may include transforming growth factor beta (TGF-*β*), nerve growth factor (NGF), and insulin-like growth factors (IGFs) and others [29]. Typically, TNF*α* is the most abundant by a large margin. Data is available on specific inflammatory factors including IL-6 and TNF*α* inducing inflammatory osteoclast formation without added RANKL [30], but likely small quantities of RANKL are present [29,31]. The inflammatory factors, particularly TNF*α*, definitely augment the effects of small amounts of RANKL [13].

This is important in inflammatory arthritis where bone degrading cells form with inflammatory cytokines that are not clearly adjacent to cells that mediate bone turnover via local RANKL expression. Indeed, RANKL knockout mice have dramatically reduced bone erosion in a serum transfer model of arthritis [32]. Further, while acknowledging the importance of TNF*α*, other cells that express RANKL including plasma cells and synovial fibroblasts are present [24,33], and are critical to osteoclast formation. That said, RANKL is an essential factor in normal bone turnover, and the importance of non-canonical pathways contributing to bone erosion in juvenile idiopathic arthritis are unclear. Nonetheless inflammatory protein targets, especially TNF*α*, are largely specific to arthritis.

RANKL is a cell membrane bound cytokine acting locally [34]. It is cleaved by proteinases and is detectable in serum. Serum concentrations of RANKL in rheumatoid arthritis average ~50 picomolar (pM) [35]. In contrast, serum TNF*α* in rheumatoid arthritis patients is much higher, averaging 5000 pM (5.0 nM) [36]. Recent work shows that TNF*α* response focuses on NF-*κ*B, overlapping the signaling of RANKL [37]. *In vivo*, when RANKL is released, it is bound, at least in large part, by the decoy receptor osteoprotegerin [34]. Osteoprotegerin also blocks RANKL signaling and is an inhibitor of bone degradation. Thus, the importance of soluble RANKL in arthritis is unclear, while the concentration of TNF*α*, a soluble protein is many times higher, and is of key importance in the pathogenesis of inflammatory arthritis.

Fig. 2 is an example from our earlier work; there are many similar reports, some with detailed cell labeling [14]. Osteoclast formation in rheumatoid arthritis clearly is not related to local bone formation, as shown in Fig. 1. The inflammatory infiltrate includes, in addition to lymphocytes and dense accumulation of macrophages and expanded synovial cells of fibroblast origin, although these are not adjacent to the tissue as demonstrated by direct labeling [14]. Thus, the infiltrate is now commonly called inflammatory synovitis.

### 2.5 Factors in Etiology of Idiopathic Arthritis Leading to Bone Destruction by Osteoclasts

What causes inflammatory arthritis is in most cases unclear.

#### 2.5.1 Historical Origin of Idiopathic, or Rheumatoid, Arthritis

Extensive analysis shows damage to joints in any population studied, but pathology reflecting chronic, non-traumatic inflammatory joint damage was rare in the old world before the European conquest of the Americas, but common in the American indigenous population (Native Americans; “American Indians”). The link of this finding specifically to inflammatory arthritis is unclear. A pathogen or allergen from the new world might have made rheumatoid arthritis much more common [38]. This topic has been discussed extensively and is controversial. However, skeletal remains suggest that rheumatoid arthritis was, at a minimum, much more common in the Americas before the European conquest [39]. This tantalizing observation suggests that at least one pathogenic or inflammatory stimulus, still unknown, might be important in triggering inflammatory arthritis. Regarding microbial agents, isolation of pathogens has been limited to the last ~150 years, so it is not clear which might have been introduced from the New World, besides syphilis. In the other direction Old World origin of important diseases including smallpox is clear [40]. The list of plants from the New World is long and important, but not clearly associated with inflammatory arthritis. That said, an obvious point is that lectins from food are aggravating factors in inflammatory arthritis; these include New World foods, an example being peanuts (Arachis hypogaea), but none are implicated as specific etiologic agents [41].

#### 2.5.2 Infectious Agents, Bacteria, Viruses, and Protozoa

These are among the clearest and earliest associations with inflammatory arthritis, indeed with such an extensive history that this is referred to other work [42]. A few systemic infections including Borrelia burgdorferi have been specifically studied and are quite interesting. Borrelia burgdorferi often causes acute inflammatory arthritis, known as Lyme disease, that may develop into chronic inflammatory arthritis [43]. Gut microbiome elements are also frequently discussed as promoting inflammatory arthritis and related diseases, as are nutrients associated with anti-inflammatory or anti-oxidant effects [44]. In addition, intestinal barrier function may similarly impact on arthritis as a secondary effect [45]. This might involve either inflammatory disease having a primary effect on intestinal barrier function, or the altered barrier function having secondary effects on joints. The role of innate immunity, including toll-like receptors and their ligands, in osteoclastogenesis may be important, although, surprisingly, stimulation of some toll-like receptors inhibits osteoclast activity with TNF*α* and might be an arthritis defense [46].

#### 2.5.3 Toxins and Arthritis

Toxins include stimuli in arthritis models, particularly, collagen induced arthritis (Fig. 2). This uses type II collagen with Freund’s adjuvant by subcutaneous injection in susceptible mouse strains. In this model, rheumatoid factor is not induced [47].

#### 2.5.4 Toxins and Genetic Predisposition

On the border of toxins is the formation of “modified self-epitopes” through any of several processes, including rheumatoid factor, anti-citrullinated protein, anti-carbamylated protein, and anti-acetylated protein antibodies [48] related to inflammatory arthritis, juvenile or rheumatoid [48]. It is established that anti-citrullinated protein antibodies (ACPA) play a key role in the pathogenesis of bone damage in rheumatoid arthritis, stimulating osteoclast differentiation, directly by binding to carbamylated proteins on surfaces of osteoclast precursors, and, indirectly, increasing the release of TNF*α* by macrophages [48]. Genetic factors predisposing to arthritis include the HLA-DRB1 shared epitope, although genetic predisposition is generally regarded as requiring a second stimulus, e.g., bacterial [49]. An open question, too complex for full consideration, is whether more aggressive management of inflammatory cytokine production, particularly TNF*α*, might in part interrupt the pathway leading to advanced disease with extensive anti-citrullinated protein antibodies.

### 2.6 Balancing Symptomatic Relief and Long Term Response in Inflammatory Arthritis

Treatments balance toxicity with efficacy, and often require that individual agents be used for limited periods and then switching to alternatives. A major question is whether to focus solely on clinical response, or to consider a balance with treatment that might also block progression effectively. With the newest inhibitors, including janus kinase inhibitors and calcium channel antagonists, it is unknown whether disease progression will be antagonized specifically.

#### 2.6.1 Monoclonal Antibodies

The occurrence of inflammatory signals, including TNF*α*, IL-6, IL-10, interferon-*γ*, and IL-17 [4,6], is discussed above. Monoclonal antibodies blocking these, including anti- TNF*α* [7], and anti-IL-6 antibodies [3], are useful in reducing or eliminating bone resorption, particularly in juvenile idiopathic arthritis [3], and have reduced the occurrence of advanced disease requiring joint replacement [8,9]. In most patients, side effects are minimal. Although elevated levels of IL-1 also occur in inflammatory arthritis, the IL-1 antagonists such as Anakinra are rarely used to treat rheumatoid arthritis in the United States because of lesser efficacy than other monoclonal antibodies. IL-1 antagonists are often withdrawn due to adverse drug reactions [50]. Additional targets with promising results include anti-IL-17 and anti-IL-17 receptor antibodies [51].

#### 2.6.2 Glucocorticoids and Methotrexate

Methotrexate is generally the first drug prescribed for adults. Initial choices in the treatment of rheumatoid arthritis overall include methotrexate together with glucocorticoid steroids (prednisone) and nonsteroidal anti-inflammatory drugs [52]. Prednisone is highly effective, but important side effects, including susceptibility to infection and osteoporosis, limit its use. In addition, in children, steroids can interfere with growth, which severely limits their use for juvenile idiopathic arthritis. In many cases, methotrexate is the best choice for activity with relatively manageable side effects [53]. While methotrexate elicits complex mechanisms, it is clear that adenosine release and trans-methylation are key activities [54]. Whether early treatment of primary changes in inflammatory cytokines might reduce progression is a point of active investigation [10].

#### 2.6.3 Anti-RANKL Therapy

It is important not to minimize the RANKL system in inflammatory arthritis. There is a complex web of growth factors, where ideally the inflammatory arthritis can be inhibited with minimal nonspecific effects, and RANKL is of course central to normal bone turnover. Specifically, RANKL is induced in response to IL-6, and TNF-*α*, IL-17, or IL-1*β* stimulate production of IL-6 in synovial fibroblasts [55]. Fibroblasts are the critical MSC-derived cells in the inflammatory infiltrate that mediate bone destruction, both in cartilage and mineralized bone. In addition, expression of RANKL in synovial cells increases in rheumatoid arthritis [56]. These findings together strongly suggested the possible increased efficacy of combined therapy, as opposed to single factor therapy directed at RANKL in inflammatory arthritis. On the other hand, a careful double-blind clinical trial of the RANKL-inhibiting monoclonal antibody denosumab showed sustained suppression of markers of bone turnover but no evidence of an effect on joint space narrowing or measures of rheumatoid arthritis disease activity [57]. This suggests that, despite the role of RANKL in osteoclast formation in inflammatory arthritis established in knockout mice [32], the inflammatory cytokines, clinically, are more important targets.

#### 2.6.4 Janus Kinase Inhibitors

Janus kinase transduces and activates protein transcription from cell surface cytokine receptors. Recently developed inhibitors variably down-regulate the effects of cytokines. They are effective against rheumatoid or juvenile arthritis and other immune-mediated inflammatory diseases [58]. As with antibodies to inflammatory cytokines, serious adverse reactions are unusual but have been reported, including anemia and lymphopenia; moreover, as with all drugs acting on the immune system, infections such as reactivation of tuberculosis are an issue [59].

#### 2.6.5 Small Molecule Inhibitors Targeting Intracellular Calcium Signals

The search for arthritis inhibitors with limited systemic toxicity has led to the pursuit of inhibitors of Orai1, the pore forming unit of the calcium release-activated calcium (CRAC) channel. This system is required for efficient osteoclast production [15], and a small molecule inhibitor of Orai1, 3,4-dichloropropionaniline, showed promising results inhibiting inflammatory arthritis induced by collagen II, with minimal effects on tissues including bone not involved with the arthritis [13]. This led to an ongoing search for analogs with better activity and still less toxicity. In this regard, N-Methyl-3,4-dichloropropionaniline is a potent inhibitor of TRPC4, the new pathway in osteoclast formation [13] and a key new small molecule candidate for treatment of inflammatory arthritis.

#### 2.6.6 TNF Inhibitors with Non-biologic Disease Modifying Anti-rheumatic Drugs

This is also a subject of active investigation. The central role of TNF inhibitors is widely acknowledged although in many cases it may be useful to include non-biological disease modifying anti-rheumatic drugs (DMARDs), e.g., [60]. Alternatively, other cytokine inhibitors, of IL-6, next in importance to TNF*α*, or others including IL-17, or anti-receptor antibodies. The role of IL-17 inhibitors in juvenile inflammatory arthritis is to block Th17 cells that produce massive tissue reaction [61].

## 3. Conclusions

Overall, greater success, for treatment of juvenile inflammatory arthritis is seen if it is treated aggressively with biological anti-cytokine antibodies. Inflammatory arthritis is mediated in major part by bone destruction involving dense mononuclear cell infiltrates with osteoclasts and osteoclast-like bone degrading cells. This is amenable, particularly in juvenile disease, to therapy treating inflammatory factors and pathways for the formation of bone-degrading cells that are major factors in arthritis but are minor factors in normal bone turnover. There are many important related disease findings, including similar inflammatory cytokines with disease related to back pain [62]. Further, it should be noted that RA has also been studied by genome wide association studies that have contributed importantly to understanding of inflammatory cytokines and factors including ethnicity [63].
